# Association Between the Korean Healthy Eating Index (KHEI) and Healthcare Costs Among Adults: The Korea National Health and Nutrition Examination Survey (KNHANES) 2016 and 2021

**DOI:** 10.3390/nu17132237

**Published:** 2025-07-06

**Authors:** Soyoung Kim, Minseon Park

**Affiliations:** 1 Department of Family Medicine, Seoul National University Hospital, Seoul National University College of Medicine, Seoul 03080, Republic of Korea; soooooy7759@gmail.com; 2 Department of Family Medicine, College of Medicine, Seoul National University, Seoul 03080, Republic of Korea

**Keywords:** diet quality, Korean Healthy Eating Index, healthcare costs, healthcare utilization, KNHANES

## Abstract

Background/Objectives: This cross-sectional study examined the association between diet quality, measured by the Korean Healthy Eating Index (KHEI), and medical expenditures among Korean adults. Methods: We used data from the Korea National Health and Nutrition Examination Survey (2016–2021). Adults aged ≥20 years with complete data on diet, sociodemographics, and healthcare use were included. Medical costs were estimated from self-reported service use and converted to USD. KHEI scores were categorized into quartiles. Multivariable linear regression was used to assess the association between KHEI quartiles and log-transformed costs. Subgroup analyses were conducted by age (<57 vs. ≥57 years), and sensitivity analyses treated KHEI as a continuous variable. A two-part model addressed skewed, zero-inflated cost data. Results: Compared to Q1, participants in Q4 had significantly lower inpatient (β = −0.080; 95% CI: −0.139 to −0.020) and total costs (β = −0.086; 95% CI: −0.144 to −0.027). In the younger group, Q4 was associated with lower total costs (β = −0.115; 95% CI: −0.198 to −0.031). Higher continuous KHEI scores were also linked to lower costs. In the two-part model, Q4 participants had a higher probability of incurring any cost but lower conditional costs (Q3 vs. Q1: β = −0.173; Q4 vs. Q1: β = −0.160; both *p* < 0.05). Conclusions: Higher diet quality was associated with reduced healthcare costs in Korean adults, especially among younger individuals. Promoting healthy eating may help lower economic burdens in aging societies.

## 1. Introduction

As population aging accelerates worldwide, medical expenditures are steadily increasing. Korea is also rapidly becoming an aging society, and the growing prevalence of chronic diseases is placing a significant burden on the National Health Insurance system. Consequently, various strategies are being explored to reduce healthcare costs.

Recently, healthy diets have gained increasing attention for their potential impact not only on disease prevention but also on healthcare expenditures. Previous studies have confirmed that food insecurity—a condition often associated with poor diet quality—is linked to increased healthcare utilization and costs [1]. Furthermore, an Australian longitudinal study of middle-aged women demonstrated that diet quality was significantly associated with cumulative healthcare expenditures over a 10-year period [2]. These findings suggest that diet quality may influence not only individual health outcomes but also the use of healthcare resources and the overall economic burden.

In Korea, the Korean Healthy Eating Index (KHEI) has been developed as a standardized tool for quantitatively assessing the quality of eating habits [3]. An increasing number of studies are utilizing the KHEI to explore associations between dietary quality and various health outcomes. For example, among middle-aged adults living in single-person households, lower KHEI scores have been associated with a higher risk of chronic conditions, including metabolic abnormalities such as hypertension and diabetes [4,5]. A nationwide study also found that poor diet quality was linked to adverse cardiometabolic outcomes in Korean adults [6]. Furthermore, a study of adults aged 40 years and older reported that lower KHEI scores were associated with increased risks of obesity and sarcopenia, and also identified regional disparities in diet quality between urban and rural areas [7].

However, most existing studies have focused on the relationship between dietary quality and health outcomes, while research on the association between dietary quality—particularly as measured by the KHEI—and actual medical expenditures remains limited in Korea. Therefore, this study aims to examine the relationship between KHEI and medical expenses and to assess whether community-based nutritional improvements can help reduce healthcare costs at the level of primary care. The results may help to demonstrate the economic benefits of healthier dietary patterns and support policy-level interventions. Ultimately, the findings may provide a foundation for developing diet-centered health management strategies and informing future healthcare resource allocation.

## 2. Materials and Methods

### 2.1. Study Design and Participants

This cross-sectional study was based on data from the 2016 to 2021 Korea National Health and Nutrition Examination Survey (KNHANES), conducted by the Korea Disease Control and Prevention Agency (KDCA). KNHANES is an annual, population-based survey that collects information on health status, behaviors, and dietary intake from non-institutionalized civilians in Korea using a stratified, multistage, clustered sampling design, making the dataset nationally representative. Further methodological details are available from the official website (http://knhanes.kdca.go.kr, accessed on 3 April 2025).

For this analysis, we included adults aged 20 years or older with complete data on dietary intake, medical utilization, and covariates of interest. To ensure valid assessment of healthcare costs, individuals with zero medical expenditures were excluded. A total of 1144 participants were included in the final analysis. To evaluate the representativeness of the included sample, we compared key characteristics between participants with and without medical expenditures. The results of this comparison are presented in Supplementary Table S1. For the sensitivity analysis using the two-part model, all individuals with complete data on dietary intake, medical utilization, and covariates were included regardless of their medical expenses (*n* = 25,194), to account for the full distribution of healthcare costs, including non-users.

Informed consent was obtained from all participants, and all data were anonymized before analysis. The study protocols of KNHANES were approved by the KDCA Institutional Review Board (IRB No.: 2018-01-03-P-A, 2018-01-03-C-A, 2018-01-03-2C-A, 2018-01-03-5C-A).

No generative AI tools were used in the design, analysis, or writing of this study beyond grammar and formatting assistance.

### 2.2. Dietary Assessment and Korean Healthy Eating Index (KHEI)

Dietary intake data were collected using a 24-h recall and a food frequency questionnaire (FFQ), both administered by trained dietitians. The 24-h recall was used to estimate daily energy and nutrient intake based on the Korean Food Composition Table.

The Korean Healthy Eating Index (KHEI), developed by the KDCA, is a composite score (range: 0–100) based on 14 components assessing adequacy, moderation, and energy balance. It was calculated using validated FFQ data and was provided as a pre-calculated variable in the KNHANES dataset. Higher scores indicate greater adherence to national dietary guidelines and healthier overall diet quality [3]. In this study, the pre-calculated KHEI score was used as the primary exposure variable.

### 2.3. Assessment of Healthcare Costs

Healthcare utilization data were obtained via self-administered questionnaires. Participants reported the number of outpatient visit days over the past two weeks and inpatient days over the past year. Medical expenditures were estimated by multiplying reported utilization by year-specific average unit costs provided by the National Health Insurance Service (NHIS) of Korea [8]. Total medical costs were the sum of outpatient and inpatient costs. All costs were converted from Korean won (KRW) to US dollars (USD) using the average exchange rate of 1 USD = 1150 KRW (2016–2021 average).

### 2.4. Covariates

Covariates were selected based on their relevance to diet quality and healthcare costs. Sociodemographic factors included age (continuous), sex (male or female), household income (low: lowest 2 quintiles vs. high: top 3 quintiles), and education (low: middle school or less vs. high: high school or more) [9,10]. Body mass index (BMI) was calculated as: BMI = weight (kg)/height (m^2^) [11]. Health status was captured using a weighted comorbidity score based on self-reported physician-diagnosed chronic conditions: hypertension, dyslipidemia, stroke, myocardial infarction, diabetes, various cancers, kidney disease, and liver cirrhosis. Cancer, kidney disease, and liver cirrhosis were assigned 2 points each; all others were assigned 1 point [12]. Lifestyle variables included smoking (never vs. ever smoker) and alcohol consumption (never vs. ever drinker), both assessed via self-report [13].

### 2.5. Statistical Analysis

Descriptive statistics summarized the study population. The normality of all con-tinuous variables was assessed using the Shapiro–Wilk test, which showed that none of the variables followed a normal distribution. Therefore, Continuous variables were expressed as medians with interquartile ranges (IQRs) and compared across KHEI quartiles using the Kruskal–Wallis test. Categorical variables were summarized as counts and percentages and compared using the chi-square test. Dunn’s test with Bonferroni correction was applied for post-hoc comparisons of medical costs across quartiles.

Multiple linear regression models assessed the association between KHEI quartiles (Q1–Q4) and log-transformed inpatient, outpatient, and total medical costs, adjusting for all covariates. Results were reported as β coefficients with 95% confidence intervals (CIs). Forest plots were used to visualize the associations. Interaction effects between KHEI and sex were tested using a cross-product term, and estimated marginal means were compared across groups.

Subgroup analyses were stratified by age based on the median value (<57 vs. ≥57 years). As a sensitivity analysis, KHEI scores were also modeled as continuous variables. To address zero-inflated, skewed cost data, a two-part model was applied: the first part used logistic regression to model the probability of any healthcare use, and the second part modeled positive expenditures using a generalized linear model with a log link [14]. These models were selected to minimize potential biases arising from violations of asymptotic normality assumptions in highly skewed data.

The assumptions of the regression models were assessed using diagnostic plots: smoothed scatter plots were used to evaluate linearity between continuous variables (e.g., age, KHEI) and log-transformed costs, and Q–Q plots of residuals were examined to check the normality of errors. All analyses were performed using R software (version 4.3.2; R Foundation for Statistical Computing, Vienna, Austria). A *p*-value < 0.05 was considered statistically significant.

## 3. Results

### 3.1. General Characteristics of the Study Populations

**Figure 1:**
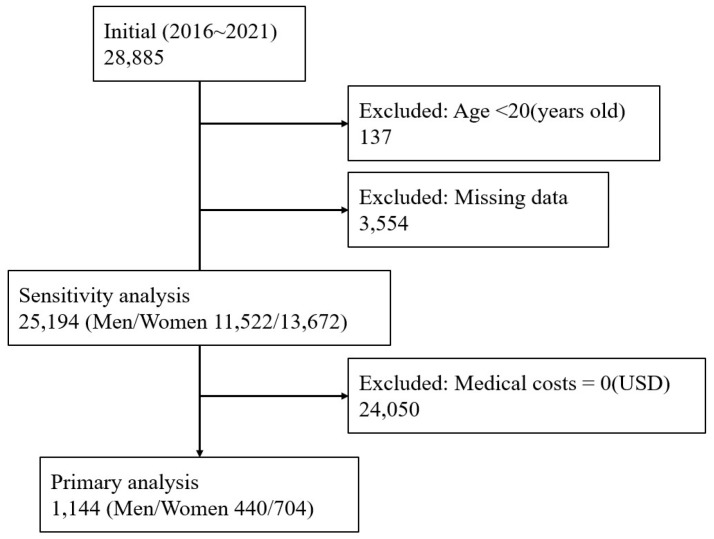
Flowchart illustrating the selection of study participants from the Korea National Health and Nutrition Examination Survey (2016–2021). Participants under 20 years of age or with missing data on key variables (e.g., diet, demographics, or medical use) were excluded. Among the remaining 25,194 adults, those with zero medical costs were excluded, resulting in a final analytic sample of 1144 adults (440 men and 704 women). Participants excluded due to zero medical costs were included in a sensitivity analysis.

A total of 1144 participants were included in the analysis after applying inclusion and exclusion criteria (Figure 1).

The median age of the study population was 57.0 years (interquartile range [IQR]: 42.0–68.0 years), and 61.5% were female. The median body mass index (BMI) was 24.18 kg/m^2^ (IQR: 21.96–26.29 kg/m^2^). Regarding socioeconomic status, 41.0% of participants were classified as having low household income, and 40.4% had a low level of educational attainment. In terms of health behaviors, 41.3% of the participants were classified as ever smokers, while 68.4% reported current alcohol consumption. The median number of chronic diseases was 1.0 (IQR: 0.0–2.0).

The median inpatient medical cost was 1295.84 USD (IQR: 1193.10–1393.09 USD), while the median outpatient medical cost was 41.65 USD (IQR: 22.87–62.47 USD). The median total medical cost was 1320.03 USD (IQR: 1231.46–1448.37 USD) (Table 1).

### 3.2. Comparison of Clinical Characteristics and Medical Costs According to KHEI Quartiles

The clinical, socioeconomic, and behavioral characteristics of participants according to Korean Healthy Eating Index (KHEI) quartiles are presented in Table 2. Participants in higher KHEI quartiles tended to be older, with the median age increasing from 47.0 years in Q1 to 61.0 years in Q4 (*p* < 0.001). The proportion of females increased across quartiles, from 61.0% in Q1 to 69.3% in Q4 (*p* = 0.003). Higher household income and higher educational attainment were significantly more prevalent in the higher KHEI quartiles (*p* = 0.004 and *p* < 0.001, respectively). The proportion of never smokers increased significantly with higher KHEI quartiles (*p* < 0.001), while alcohol consumption did not significantly differ across groups (*p* = 0.062). The number of chronic diseases also increased with higher KHEI scores (*p* < 0.001).

Regarding healthcare costs, participants in the highest KHEI quartile (Q4) showed significantly lower inpatient and total medical costs compared to those in the lowest quartile (Q1). The median inpatient cost decreased from 1295.84 USD in Q1 to 1209.77 USD in Q4 (*p* = 0.007), and the median total medical cost decreased from 1347.81 USD to 1319.13 USD (*p* = 0.005), while outpatient costs did not differ significantly (*p* = 0.395).

Post-hoc pairwise comparisons using Dunn’s test with Bonferroni correction revealed that the significant differences in costs were mainly between Q1 and Q4 for both inpatient (*p* = 0.004) and total medical costs (*p* = 0.002) (Table 3).

**Figure 2:**
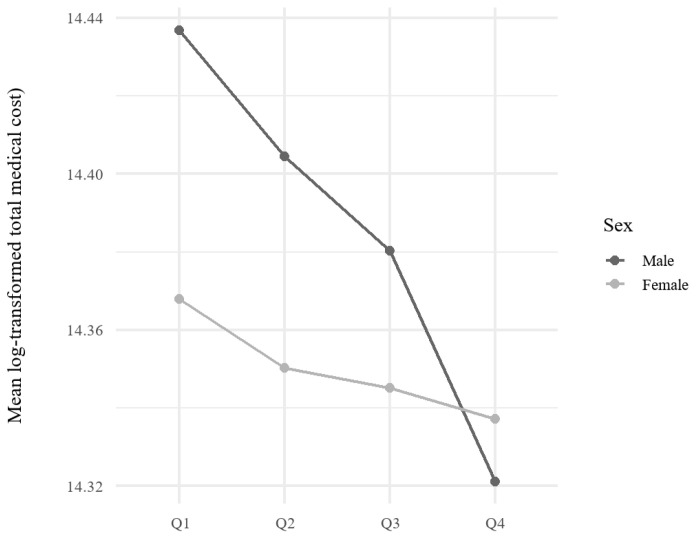
Interaction plot of KHEI quartiles and sex on mean log-transformed total medical costs. This figure illustrates the relationship between KHEI quartiles and total medical costs, stratified by sex. Mean log-transformed total medical costs are plotted for each KHEI quartile for men and women separately.

An interaction analysis was performed to investigate whether the association between KHEI quartiles and total medical costs differed by sex. The interaction terms were not statistically significant (*p* for interaction > 0.05), suggesting that the relationship between KHEI and total medical costs was consistent across sexes (Figure 2).

### 3.3. Association Between KHEI and Medical Costs

**Figure 3:**
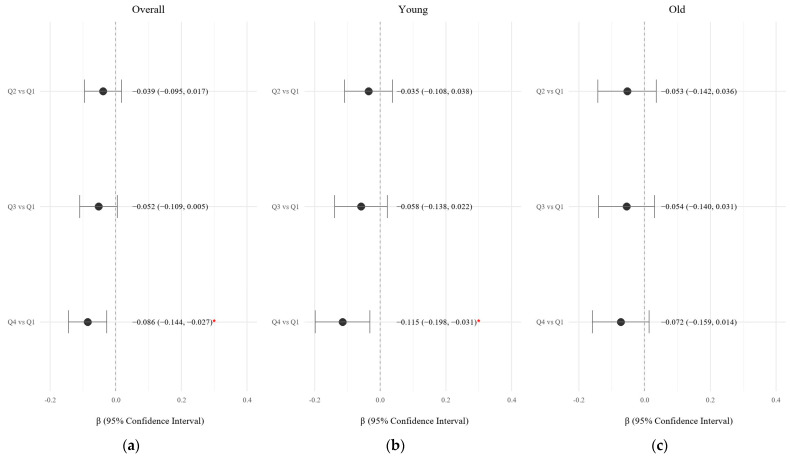
Forest plot of the associations between KHEI quartiles and log-transformed total medical costs, stratified by age: (**a**) Overall population (**b**) Younger group (<57 years) (**c**) Older group (≥57 years). The figure presents β coefficients and 95% confidence intervals (CIs) from multivariable linear regression models comparing KHEI quartiles (Q2–Q4) to Q1 (reference group). Estimates are shown for the overall population, younger adults (<57 years), and older adults (≥57 years). Models were adjusted for age (in overall analysis), sex, household income, education level, smoking status, alcohol use, body mass index (BMI), and number of chronic diseases. Red points indicate statistically significant associations (* *p*-value < 0.05). β = beta coefficient; CI = confidence interval.

Multivariable linear regression analyses were conducted to evaluate the association between KHEI quartiles and log-transformed medical costs, adjusting for potential confounders including age, sex, income group, education level, number of chronic diseases, BMI, smoking, and alcohol consumption (Table 4, Figure 3). Compared to Q1, participants in Q4 had significantly lower inpatient costs (β = −0.080; 95% CI: −0.139 to −0.020; *p* = 0.009) and total medical costs (β = −0.086; 95% CI: −0.144 to −0.027; *p* = 0.004). Outpatient costs were also significantly lower among participants in Q4 compared to Q1 (β = −0.121; 95% CI: −0.238 to −0.003; *p* = 0.045).

In age-stratified analyses, the inverse association between KHEI quartiles and total medical costs remained statistically significant in the younger group (Q4 vs. Q1: β = −0.115; 95% CI: −0.198 to −0.031), but not in the older group (Figure 3).

In sensitivity analyses treating KHEI as a continuous variable, higher KHEI scores were associated with significantly lower inpatient costs (β = −0.002; 95% CI: −0.004 to −0.001; *p* = 0.003) and total medical costs (β = −0.003; 95% CI: −0.004 to −0.001; *p* = 0.001), whereas the association with outpatient costs did not reach statistical significance (*p* = 0.061).

**Figure 4:**
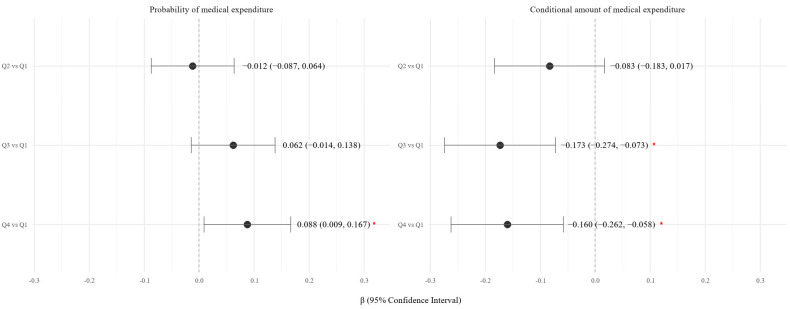
Forest Plot of the Association Between KHEI Quartile and Total Medical Expenditure Using a Two-Part Model. The left panel shows the β coefficients and 95% confidence intervals (CIs) for the association between KHEI quartiles and the probability of incurring any medical expenditure (logistic regression). The right panel presents β coefficients for the association between KHEI quartiles and the amount of expenditure among individuals with non-zero costs (generalized linear model with a log link). All models were adjusted for sex, age, income, education, smoking, alcohol use, BMI, and number of chronic diseases. Red * indicate statistically significant associations (*p*-value < 0.05). Q1 (lowest quartile) was used as the reference group. β = beta coefficient; CI = confidence interval.

Additionally, to address the skewness and zero inflation of medical cost data, a two-part model was applied (Figure 4). The first part used logistic regression to assess the probability of incurring any medical expenditure, and the second part used gamma regression to estimate the amount of expenditure among those with positive costs. Compared to Q1, participants in Q4 had a significantly higher probability of having any medical expenditure (β = 0.088; 95% CI: 0.009 to 0.167; *p* < 0.05). Furthermore, participants in Q3 and Q4 had significantly lower medical expenditures among spenders (Q3 vs. Q1: β = −0.173; 95% CI: −0.274 to −0.073; *p* < 0.05; Q4 vs. Q1: β = −0.160; 95% CI: −0.262 to −0.058; *p* < 0.05).

## 4. Discussion

In this study, we found that higher adherence to a healthy dietary pattern, as assessed by the Korean Healthy Eating Index (KHEI), was significantly associated with lower healthcare expenditures among Korean adults. Participants with higher KHEI scores had reduced inpatient and total medical costs compared to those with lower scores, even after adjusting for potential confounders such as age, sex, socioeconomic status, comorbidities, BMI, smoking, and alcohol consumption.

Previous studies have demonstrated that a healthy diet reduces the risk of chronic diseases including cardiovascular disease, diabetes, and cancer—major contributors to healthcare costs [4,6,15]. Our findings expand on this evidence by showing that overall diet quality, rather than individual nutrients or food items, is inversely associated with medical expenditures.

Although participants with higher KHEI scores were older and had a greater burden of chronic conditions, they still incurred lower total and inpatient medical costs. This counterintuitive result may be explained by better disease management among individuals with healthier diets. Healthier dietary habits may lead to fewer complications and hospitalizations despite the presence of chronic diseases.

In unadjusted comparisons using the Kruskal–Wallis test, differences in inpatient costs between KHEI quartiles were not statistically significant. However, multivariable models adjusting for key covariates revealed significant associations. This highlights the importance of controlling for confounding factors when examining the relationship between diet and medical costs.

Notably, in age-stratified analyses, the inverse association between KHEI and total medical costs remained significant only in the younger group (<57 years), with a larger effect size compared to the older group. One possible explanation is that younger adults may be more susceptible to poor dietary habits such as meal skipping or processed food consumption—behaviors linked to chronic disease risk and increased healthcare use. In contrast, older adults may be more vulnerable to malnutrition due to imbalanced intake, leading to acute issues such as frailty, falls, or infections. These conditions may increase medical costs despite better overall diet quality. Furthermore, although the KHEI is a comprehensive index, it may not fully reflect micronutrient deficiencies (e.g., calcium, vitamin D) that are particularly important in older adults [3,16,17,18,19]. This limitation could partially explain the weaker association observed in this age group. These findings suggest that the mechanisms linking diet quality and healthcare costs may differ by age, and tailored interventions may be needed.

To address the skewed and zero-inflated distribution of medical cost data, we applied a two-part model. The first part evaluated the likelihood of incurring any medical expenditure, and the second part modeled the conditional amount of spending among those who did incur costs. Compared to Q1, participants in Q4 had a significantly higher probability of using healthcare services. However, among users, participants in Q3 and Q4 spent significantly less. These findings suggest that individuals with healthier diets may engage more in preventive or outpatient care, leading to reduced need for intensive treatment and overall cost savings.

The major strength of this study lies in its use of a large, nationally representative sample with detailed dietary and healthcare cost data. The application of both conventional regression models and a two-part model enhances the robustness of our findings and accounts for the specific distributional characteristics of cost data.

Several limitations should be noted. First, the cross-sectional design limits causal inference. Second, dietary intake was assessed using a single 24-h recall, which may not reflect usual intake. Third, medical expenditures were self-reported over different time frames (two weeks for outpatient, twelve months for inpatient), potentially introducing recall bias. Fourth, although we adjusted for multiple confounders, unmeasured factors such as genetic predisposition, healthcare accessibility, or health-seeking behavior may have influenced the results. Fifth, individuals with zero medical costs were excluded from the primary analysis. Although this approach allowed for more accurate estimation of expenditure amounts, it may have introduced selection bias. A comparison between included and excluded participants revealed that those included in the analysis were older, had higher BMI and more chronic conditions, and were more likely to be female and less educated. These differences suggest that our findings may be more reflective of individuals with greater healthcare needs, and caution is warranted in generalizing results to the entire adult population. Lastly, these findings may not be generalizable to countries with different healthcare systems, cost structures, or dietary patterns.

In summary, higher KHEI scores were associated with lower healthcare expenditures, particularly among younger adults. Although the trend was similar in older adults, the association was not statistically significant. These findings suggest that promoting healthy eating from early adulthood may help reduce future medical costs. Longitudinal studies are needed to confirm these findings.

## Figures and Tables

**Table 1:** Clinical characteristics of the study population. Continuous variables are presented as medians with interquartile ranges [IQR], and categorical variables are expressed as number (percentage). Chronic diseases include self-reported diagnoses such as hypertension, dyslipidemia, stroke, myocardial infarction, diabetes, various cancers, kidney disease, and liver cirrhosis. Medical costs are estimated in US dollars ($) based on self-reported healthcare utilization. BMI = body mass index.

Variables	Unweighted (N = 1144)
Continuous variabales, Median [IQR]	
Age	57.00 [42.00, 68.00]
BMI	24.18 [21.96, 26.29]
Number of Chronic diseases	1.00 [0.00, 2.00]
Medical costs ($)	
Inpatient costs	1295.84 [1193.10, 1393.09]
Outpatient costs	41.65 [22.87, 62.47]
Total medical costs	1320.03 [1231.46, 1448.37]
Categorical variables, N (%)	
Sex	
Male	440 (38.5)
Female	704 (61.5)
Income	
Low	469 (41.0)
High	675 (59.0)
Education	
Low	462 (40.4)
High	682 (59.6)
Smoking	
Never	671 (58.7)
Ever	473 (41.3)
Alcohol drinking	
Never	361 (31.6)
Ever	783 (68.4)

**Table 2:** Characteristics of the study population according to Korean Healthy Eating Index quartiles. Continuous variables are presented as median [interquartile range] and were compared across KHEI quartiles using the Kruskal–Wallis test. Categorical variables are expressed as number (percentage) and were compared using the chi-square test. Medical costs are presented in US dollars ($). * *p*-value < 0.05. BMI = body mass index.

	Korean Healthy Eating Index				
	Q1 [18.14–53.55]	Q2 [53.55–63.09]	Q3 [63.09–72.12]	Q4 [72.12–98.20]	*p*-value
Unweighted N	272	290	292	290	
Continuous variables, Median [IQR]					
Age	47.00 [34.00, 61.00]	56.00 [39.00, 69.00]	60.00 [47.00, 71.00]	61.00 [50.00, 69.00]	<0.001 *
BMI	24.24 [21.81, 26.60]	24.14 [21.76, 26.51]	24.37 [22.56, 26.25]	23.83 [21.70, 26.05]	0.287
Number of Chronic diseases	0.00 [0.00, 2.00]	1.00 [0.00, 2.00]	1.00 [0.00, 2.00]	1.00 [0.00, 2.00]	<0.001 *
Medical costs ($)					
Inpatient costs	1295.84 [1209.77, 1393.09]	1295.84 [1193.10, 1393.09]	1295.84 [1193.10, 1393.09]	1209.77 [1193.10, 1322.09]	0.007 *
Outpatient costs	27.64 [22.87, 62.47]	41.65 [24.19, 62.47]	41.65 [24.19, 65.08]	27.64 [22.87, 51.44]	0.395
Total medical costs	1347.81 [1248.97, 1746.41]	1344.23 [1234.74, 1448.37]	1320.03 [1234.74, 1441.50]	1319.13 [1231.46, 1420.73]	0.005 *
Categorical variables, N (%)					
Sex					0.003 *
Male	106 (39.0)	111 (38.3)	134 (45.9)	89 (30.7)	
Female	166 (61.0)	179 (61.7)	158 (54.1)	201 (69.3)	
Income					0.004 *
Low	124 (45.6)	136 (46.9)	109 (37.3)	100 (34.5)	
High	148 (54.4)	154 (53.1)	183 (62.7)	190 (65.5)	
Education					<0.001 *
Low	81 (29.8)	118 (40.7)	132 (45.2)	131 (45.2)	
High	191 (70.2)	172 (59.3)	160 (54.8)	159 (54.8)	
Smoking					<0.001 *
Never	136 (50.0)	162 (55.9)	161 (55.1)	212 (73.1)	
Ever	136 (50.0)	128 (44.1)	131 (44.9)	78 (26.9)	
Alcohol Drinking					0.062
Never	69 (25.4)	91 (31.4)	99 (33.9)	102 (35.2)	
Ever	203 (74.6)	199 (68.6)	193 (66.1)	188 (64.8)	

**Table 3:** Post-hoc pairwise comparisons of medical costs between KHEI quartiles using Dunn’s test with Bonferroni correction. Pairwise comparisons of inpatient, outpatient, and total medical costs were conducted across KHEI quartiles following a significant Kruskal–Wallis test result. Adjusted *p*-values were calculated using Dunn’s test with Bonferroni correction. * *p*-value < 0.05.

Inpatient Costs		Outpatient Costs		Total Medical Costs	
Comparison	*p*-Value	Comparison	*p*-Value	Comparison	*p*-Value
Q1–Q2	1.000	Q1–Q2	1.000	Q1–Q2	0.896
Q1–Q3	0338	Q1–Q3	1.000	Q1–Q3	0.507
Q1–Q4	0.004 *	Q1–Q4	1.000	Q1–Q4	0.002 *
Q2–Q3	1.000	Q2–Q3	1.000	Q2–Q3	1.000
Q2–Q4	0.172	Q2–Q4	0.986	Q2–Q4	0.186
Q3–Q4	0.746	Q3–Q4	0.710	Q3–Q4	0.364

**Table 4:** Multivariable linear regression for log-transformed medical costs according to KHEI quartiles. Regression models were adjusted for age, sex, household income, education level, smoking status, alcohol consumption, body mass index (BMI), and the number of chronic diseases. KHEI Q1 (lowest quartile) was used as the reference group. β estimates and 95% confidence intervals (CIs) are presented for each outcome (inpatient, outpatient, and total medical costs). All cost variables were log-transformed. * *p*-value < 0.05.

KHEI Quartiles (vs. Q1)	β (Estimate)	95% CI	*p*-Value
Inpatient Costs			
Q2	−0.033	(−0.090, 0.024)	0.257
Q3	−0.050	(−0.108, 0.009)	0.100
Q4	−0.080	(−0.139, −0.020)	0.009 *
Outpatient costs			
Q2	−0.028	(−0.141, 0.085)	0.626
Q3	−0.028	(−0.143, 0.088)	0.636
Q4	−0.121	(−0.238, −0.003)	0.045 *
Total medical costs			
Q2	−0.039	(−0.095, 0.017)	0.175
Q3	−0.052	(−0.109, 0.005)	0.073
Q4	−0.086	(−0.144, −0.027)	0.004 *

## Data Availability

http://knhanes.kdca.go.kr (accessed on 3 April 2025).

## Supplementary Materials

The following supporting information can be downloaded at: https://www.mdpi.com/article/10.3390/nu17132237/s1, Table S1. Comparison of baseline characteristics between participants with zero and non-zero medical costs. Values are presented as median [interquartile range] for continuous variables and number (percentage) for categorical variables. Continuous variables were compared using the Wilcoxon rank-sum test, and categorical variables were compared using the chi-square test. *p*-values less than 0.05 were considered statistically significant. Zero medical costs: participants with total medical cost equal to 0 USD. Non-zero medical costs: participants with total medical cost greater than 0 USD.

## Abbreviations

The following abbreviations are used in this manuscript:

KHEI	Korean Healthy Eating Index
