# Supplementary material for: Association Between the Korean Healthy Eating Index (KHEI) and Healthcare Costs Among Adults: The Korea National Health and Nutrition Examination Survey (KNHANES) 2016 and 2021
Source: Nutrients. 2025 Jul 6;17(13):2237. doi: 10.3390/nu17132237 (PMC12251669; doi:10.3390/nu17132237)
Supplement: Supplementary file 1 [file nutrients-17-02237-s001.zip › nutrients-3724742-supplementary.pdf]

Table S1. Comparison of baseline characteristics between participants with zero and non-zero medical costs. Values are presented as median [interquartile range] for continuous variables and number (percentage) for categorical variables. Continuous variables were compared using the Wilcoxon rank-sum test, and categorical variables were compared using the chi-square test. P-values less than 0.05 were considered statistically significant. Zero medical costs: participants with total medical cost equal to 0 USD. Non-zero medical costs: participants with total medical cost greater than 0 USD.

	Zero medical costs	Non-zero medical costs	P-value
Unweighted N	24,050	1,144	
Continuous variables, Median [IQR]			
Age	50.00 [38.00, 63.00]	57.00 [42.00, 68.00]	<0.001*
BMI	23.67 [21.47, 26.01]	24.18 [21.96, 26.29]	<0.001*
Number of Chronic diseases	0.00 [0.00, 1.00]	1.00 [0.00, 2.00]	<0.001*
Categorical variables, N (%)			
Sex			<0.001*
Male	11,082 (46.1)	440 (38.5)	
Female	12,968 (53.9)	704 (61.5)	
Income			0.175
Low	9,367 (38.9)	469 (41.0)	
High	14,683 (61.1)	675 (59.0)	
Education			<0.001*
Low	6,176 (25.7)	462 (40.4)	
High	17,874 (74.3)	682 (59.6)	
Smoking			0.540
Never	13,875 (57.7)	671 (58.7)	
Ever	10,175 (42.3)	473 (41.3)	
Alcohol Drinking			<0.001*
Never	4,799 (20.0)	361 (31.6)	
Ever	19,251 (80.0)	783 (68.4)	
